# Importance of FISH genetics in light chain amyloidosis

**DOI:** 10.18632/oncotarget.21052

**Published:** 2017-09-19

**Authors:** Morie A. Gertz, Angela Dispenzieri, Eli Muchtar

**Affiliations:** Morie A. Gertz: Division of Hematology, Mayo Clinic, Rochester, MN, USA

**Keywords:** light chain amyloidosis, bortezomib, fluorescent *in situ* hybridization, individualized cancer treatment

Our group led by Muchtar reported on the significance of fluorescent in situ hybridization (FISH) in light chain amyloidosis patients treated both with conventional chemotherapy and high-dose therapy with stem cell transplantation. We reported a lower deep response rate in patients with t(11;14) that were treated with both bortezomib and immunomodulatory-based regimens when compared to those lacking t(11;14). This finding translated to an inferior overall survival in bortezomib and immunomodulatory-treated patients, and this adverse impact is confirmed in multivariable analysis [1]. This finding strongly suggests that patients with this specific genetic abnormality preferentially receive high-dose therapy with stem cell transplant or melphalan-based chemotherapy, which appears to abrogate this adverse effect. We also note that trisomies are a poor prognostic feature in AL amyloidosis. Abnormal cIg-FISH was significantly associated with advanced cardiac involvement and remained a prognostic factor on multivariate analysis. These findings expand and confirm a report from the Heidelberg Amyloidosis Center [2]. In that study, 101 AL amyloidosis patients uniformly treated with bortezomib-dexamethasone had FISH analysis. Unlike our population, none received high-dose chemotherapy and stem cell transplantation. There was a lower response rate, shorter progression-free and overall survival in patients with t(11;14).

Translocation (11;14) is common in plasma cell disorders, occurring in approximately 17% of patients with multiple myeloma but in 49-60% of light chain amyloidosis patients [3]. Although t(11;14) is considered a standard-risk feature in multiple myeloma, it appears that it bears an inferior outcome compared to other standard-risk markers. Translocation (11;14) in both myeloma and amyloidosis is associated with lower response rates but requires further research to understand the mechanism. It is unclear, given its high prevalence in AL amyloidosis, if t(11;14) is advantageous to the survival of the amyloid clone. Despite the disruption of the IgH locus by this translocation, it does not result in higher production of free light chain compared to AL amyloidosis patients lacking this translocation.

The importance of FISH cytogenetics in the management of multiple myeloma is well-defined, and these genetics have now been incorporated into a Revised International Staging System where the presence of t(4;14), del(17p) and/or t(14;16) are associated with higher risk and define Revised International Stage 3 if the serum β2 microglobulin is >5.5 mg/L [4]. These myeloma patients have a five-year progression-free survival of only 24%. Genetic profiling is also used in the mSMART.org risk classification for multiple myeloma. Genetics are used to define the type of maintenance therapy, preferring bortezomib with high-risk FISH features, the duration of maintenance, and the use of tandem transplantation in eligible patients with high-risk FISH genetics. Deletion 17p carries the greatest impact on outcomes in myeloma, and it has been demonstrated that the administration of bortezomib before and after autologous stem cell transplantation significantly improves outcomes in patients with this genetic abnormality [5]. The FISH findings are not only prognostic but have important therapeutic implications. Even in MGUS (where a routine marrow examination is not indicated) and smoldering multiple myeloma, FISH abnormalities are predictive of risk of progression to myeloma although are not an indication for intervention. Other hematologic malignancies also have therapy driven by the presence of genetic mutations. In chronic myelogenous leukemia, the T315I mutation in tyrosine kinase predicts resistance to all tyrosine kinase inhibitors with the exception of Ponatinib [6].

As multiple myeloma and amyloidosis are increasingly recognized to be multiclonal diseases with heterogeneous genetic profiles by gene expression profiling and by whole exome sequencing, the ability to develop individualized cancer therapies is becoming a reality. Personalized therapy in multiple myeloma is not only driven by genetics as outlined in mSMART.org but by frailty indices calculated according to patient age and vulnerability with specific guidelines now developed on the dosing of thalidomide, bortezomib, and lenalidomide in patients who are fit, frail, or intermediate. The incorporation of disability and comorbidities has become an important consideration for the clinical treatment of elderly patients with multiple myeloma. A similar approach is utilized in the AL amyloidosis population which is oftentimes frail as a result of organ impairment and requires a multidisciplinary approach to coordinate their care.

Recently, trials on the use of Venetoclax, an oral B cell lymphoma (BCL)-2 protein inhibitor, has shown specific activity in the myeloma subset of patients with the t(11;14) and heralds a new era of therapy selection specifically based on FISH abnormalities [7]. Hopefully, these results will be reproduced in the AL population, where t(11;14) is far more prevalent. The finding that FISH abnormalities drive therapy selection in light chain amyloidosis as well as myeloma is not a surprise and will certainly be followed by other treatment-specific targeted endpoints.
